# In This Issue

**Published:** 2006

## OVERVIEW: HOW IS ALCOHOL METABOLIZED BY THE BODY?

As alcohol is broken down (or metabolized) by the body it generates a number of potentially harmful byproducts. Those byproducts may lead to tissue damage, impairment of other metabolic processes, even cancer and interaction with medications. In this article, Dr. Samir Zakhari describes the various pathways of alcohol metabolism, the enzymes involved and their genetic variations, as well as the effects of alcohol metabolism and its byproducts on tissues and organs. Further research is needed to clarify the mechanisms and effects of alcohol metabolism; this will lead to intervention strategies that may help prevent its harmful effects. (pp. 245–255)

## ROLE OF ACETALDEHYDE IN MEDIATING THE PHARMACOLOGICAL AND BEHAVIORAL EFFECTS OF ALCOHOL

When alcohol is metabolized in the body, it is first broken down to acetaldehyde. Acetaldehyde accumulation outside the brain accounts for some of the adverse effects often associated with drinking, including nausea and a “flushing response.” In addition, acetaldehyde may mediate some of alcohol’s effects on the brain, although the extent of this activity is controversial. Animal studies suggest that low acetaldehyde levels in the brain may cause some of the same stimulating and reinforcing effects as does alcohol consumption. Moreover, as with alcohol, higher acetaldehyde levels have sedative effects. Some scientists therefore have proposed that acetaldehyde, rather than ethanol itself, produces many of the effects associated with drinking. Others, however, have argued that acetaldehyde levels in the brain are negligible during normal alcohol consumption, even though the alcohol-metabolizing enzyme catalase may produce acetaldehyde in parts of the brain. Based on the available evidence, Dr. Etienne Quertemont and Mr. Vincent Didone suggest that acetaldehyde likely has some direct effects on the brain and mediates some of the effects observed after alcohol consumption; however, more research is needed, especially to determine actual acetaldehyde concentrations in the brain during normal drinking. (pp. 258–265)

## OXIDATION OF ETHANOL IN THE BRAIN AND ITS CONSEQUENCES

Several studies suggest that acetaldehyde, a toxic byproduct of alcohol metabolism, may cause at least some of the behavioral effects of alcohol consumption. However, it is unclear whether quantities of acetaldehyde in the brain are sufficient to create these effects. Blood levels of acetaldehyde are very low during drinking, and, even when they are significant, acetaldehyde has difficulty penetrating the brain. This is because a unique barrier of cells protects the brain from harmful substances in the blood stream. Additionally, subjects become intoxicated even when acetaldehyde production is inhibited. However, these considerations become irrelevant if it can be shown that acetaldehyde is produced within the brain. Drs. Richard Deitrich, Sergey Zimatkin, and Sergey Pronko present evidence that alcohol is in fact metabolized to acetaldehyde in the brain and that acetaldehyde is responsible for at least some of alcohol’s behavioral effects. (pp. 266–273)

## ALCOHOL METABOLISM’S DAMAGING EFFECTS ON THE CELL

The enzyme cytochrome P450 2E1 (CYP2E1) is responsible for the metabolism of many compounds into toxic byproducts. CYP2E1’s use of oxygen in alcohol metabolism generates reactive oxygen species (ROS), which are highly reactive molecules that can cause damage to cells and tissues. This article by Dr. Dennis R. Koop examines the significant role CYP2E1 plays in generating ROS, leading to cell and tissue damage. Understanding CYP2E1’s role in alcohol metabolism and the generation of ROS is important to ultimately understanding alcohol-related tissue damage. (pp. 274–280)

## COMBINED EFFECTS OF ALDEHYDE DEHYDROGENASE VARIANTS AND MATERNAL MITOCHONDRIAL GENES ON ALCOHOL CONSUMPTION

Acetaldehyde, a toxic byproduct of alcohol metabolism, can cause a number of negative physical reactions— including feelings of nausea and the flushing response. Acetaldehyde typically is converted quickly to the nontoxic acetate in certain structures (i.e., the mitochondria) of liver cells. This conversion is mediated by the enzyme aldehyde dehydrogenase (ALDH). The faster and more efficiently ALDH reacts with acetaldehyde and other molecules, the faster acetaldehyde is removed from the body. In this article, Dr. Yedy Israel, Ms. María E. Quintanilla, Dr. Amalia Sapag, and Dr. Lutske Tampier discuss two rat strains—the alcohol-abstaining UChA rats and the alcohol-drinking UChB rats—that differ in the ALDH variants they carry as well as in the activity of their mitochondria. These animals were used to study the impact of ALDH and mitochondrial activity on alcohol metabolism and alcohol consumption. The findings suggest that mitochondrial activity during alcohol metabolism can regulate alcohol consumption not only in rats but in humans as well. (pp. 281–285)

## STUDYING ALCOHOL ELIMINATION USING THE ALCOHOL CLAMP METHOD

Investigators studying the effects of alcohol metabolism must contend with a number of potentially confounding factors, such as the subject’s gender, ethnicity, genetic variations in alcohol-metabolizing enzymes, and food consumption. One method that is helping researchers gain a better understanding of how alcohol is metabolized is the alcohol clamp method, in which alcohol is given intravenously. With this method, study participants are able to achieve and maintain a target breath alcohol concentration (BrAC) for an extended period of time. In this article, Drs. Vijay A. Ramchandani and Sean O’Connor discuss the advantages of the alcohol clamp method, which uses a computer model to estimate each individual’s specific alcohol elimination rate based on each subject’s age, height, weight, and gender. The alcohol clamp results in similar breath alcohol exposures in every subject and provides a more direct assessment of alcohol metabolism in a steady state than can be achieved by oral administration. (pp. 286–290)

## USE OF CULTURED CELLS TO STUDY ALCOHOL METABOLISM

Cells that are grown in a laboratory (i.e., cultured cells) are an important tool in studying how alcohol damages the liver on a molecular level. Alcohol metabolism results in several byproducts, and any of these byproducts, or an interaction between two or more of these byproducts, might damage the liver. Cultured cells allow researchers to investigate individual metabolic pathways, to control the cells’ exposure to alcohol and its byproducts, to eliminate variables that might confound the experiment, and to work with uniform (i.e., clonal) cells. Additionally, because large quantities of cells can be cloned, researchers are able to repeat experiments many times in order to confirm findings. Dr. Dahn L. Clemens describes important findings that have been made possible by cultured cells and explores the strengths and weaknesses of this research technique. (pp. 291–295)

## THE ROLE OF NUTRITIONAL THERAPY IN ALCOHOLIC LIVER DISEASE

The principal cause of alcoholic liver injury has been shown to be excessive alcohol consumption; however, malnutrition is closely associated with the development of alcoholic liver disease (ALD). Alcoholic patients may suffer from malnutrition because they substitute calories from food with calories from alcoholic beverages, because alcohol consumption interferes with the absorption of nutrients from the gut, and because alcohol may alter the liver’s metabolism of sugar, fats, and proteins. Drs. Christopher M. Griffith and Steven Schenker suggest that malnutrition may contribute to some of the complications associated with ALD and that nutrition repletion may improve some of these complications, particularly the increased risk of infection associated with ALD. However, nutrition alone generally does not improve survival rates; therefore, it is best administered in conjunction with other treatments. (pp. 296–306)

